# Oligoclonal expansion of TCR *V*δ T cells may be a potential immune biomarker for clinical outcome of acute myeloid leukemia

**DOI:** 10.1186/s13045-016-0353-3

**Published:** 2016-11-18

**Authors:** Zhenyi Jin, Qiang Luo, Shuai Lu, Xinyu Wang, Zifan He, Jing Lai, Shaohua Chen, Lijian Yang, Xiuli Wu, Yangqiu Li

**Affiliations:** 1 Institute of Hematology, Medical College, Jinan University, Guangzhou, 510632 China; 2 Department of Hematology, First Affiliated Hospital, Jinan University, Guangzhou, 510632 China; 3 Key Laboratory for Regenerative Medicine of Ministry of Education, Jinan University, Guangzhou, 510632 China

**Keywords:** Acute myeloid leukemia, γδ T cells, T cell receptor, Clonality

## Abstract

**Background:**

Recent data have shown that γδ T cells can act as mediators for immune defense against tumors. Our previous study has demonstrated that persisting clonally expanded *TRDV4* T cells might be relatively beneficial for the outcome of patients with T cell acute lymphoblastic leukemia after hematopoietic stem cell transplantation (HSCT). However, little is known about the distribution and clonality of the *TRDV* repertoire in T cell receptor (TCR) of γδ T cells and their effects on the clinical outcome of patients with acute myeloid leukemia (AML). The aim of this study was to assess whether the oligoclonal expansion of TCR *V*δ T cells could be used as an immune biomarker for AML outcome.

**Findings:**

γδ T cells were sorted from the peripheral blood of 30 patients with untreated AML and 12 healthy donors. The complementarity-determining region 3 (CDR3) sizes of eight TCR *V*δ subfamily genes (*TRDV1* to *TRDV8*) were analyzed in sorted γδ T cells using RT-PCR and GeneScan. The most frequently expressed *TRDV* subfamilies in the AML patients were *TRDV8* (86.67 %) and *TRDV*2 (83.33 %), and the frequencies for *TRDV1*, *TRDV3*, *TRDV4*, and *TRDV6* were significantly lower than those in healthy individuals. The most frequent clonally expanded *TRDV* subfamilies in the AML patients included *TRDV8* (56.67 %) and *TRDV4* (40 %). The clonal expansion frequencies of the *TRDV2* and *TRDV4* T cells were significantly higher than those in healthy individuals, whereas a significantly lower *TRDV1* clonal expansion frequency was observed in those with AML. Moreover, the oligoclones of *TRDV4* and *TRDV8* were independent protective factors for complete remission. Furthermore, the oligoclonal expansion frequencies of *TRDV5* and *TRDV6* in patients with relapse were significantly higher than those in non-recurrent cases.

**Conclusions:**

To the best of our knowledge, we characterized for the first time a significant alteration in the distribution and clonality of the *TRDV* subfamily members in γδ T cells sorted from AML patients. Clonally expanded *TRDV4* and *TRDV8* T cells might contribute to the immune response directed against AML, while oligoclonal *TRDV5* and *TRDV6* might occur in patients who undergo relapse. While the function of such γδ T cell clones requires further investigation, *TRDV* γδ T cell clones might be potential immune biomarkers for AML outcome.

**Electronic supplementary material:**

The online version of this article (doi:10.1186/s13045-016-0353-3) contains supplementary material, which is available to authorized users.

## Introduction

Acute myeloid leukemia (AML) is a fast-growing malignant hematological disease that occurs in large, immature white blood cells [1]. The immune systems of patients with AML become uncontrolled, leading to leukemia that cannot develop normal-functioning blood cells. Although treatments for curing AML, such as chemotherapy and hematopoietic stem cell transplantation (HSCT), have appeared in recent years, the outcome of some patients who are unable to undergo intensive chemotherapy and HSCT remains dismal with a poor survival of only 5 to 10 months [2, 3]. Therefore, novel strategies such as cellular immunotherapy have been proposed and increasingly investigated.

In the past decade, there have been numerous efforts toward developing specific T cell-based immunotherapies to manage cancer [4–6]. γδ T cells are a T cell subset that comprise approximately 5–10 % of all peripheral T cells in healthy individuals [7]. Due to the antitumor function of γδ T cells, they have been proposed to have therapeutic potential for cancer treatment [8–10]. Several in vivo and in vitro data have demonstrated that γδ T cells are excellent candidates for further improving immunotherapy efficacy because of their intrinsic characteristics and function [11, 12]. Accumulating evidence supports a particular antitumor cytotoxicity value for γδ T cells in the development of immunotherapy-based approaches for hematological malignancies such as myelodysplastic syndromes (MDS), multiple myeloma (MM), and chronic myeloid leukemia (CML) [13–15]. Despite encouraging preclinical studies of some hematological malignancies, γδ T cell-based immunotherapy for AML patients remains in its infancy, and the immune characteristics of γδ T cells in AML require further elucidation.

Recent insights into the structure of the γδ T cell receptor (TCR) and its ligands strongly indicate that γδ T cells possess unique functions for defending hosts against an extensive range of infections and stresses [7, 10, 16]. A growing body of evidence demonstrates that γδ T cells can act as functional agents for immune defense against tumors or pathogenic invaders in inflammatory reactions; they perform different functions based on their tissue distribution, antigen–receptor structure, and local microenvironment [17]. Recently, it has been reported that the phenotype and distribution of γδ T cells in human breast cancer might serve as a prognostic factor predicting clinical outcome [18]. Our previous study reported that clonally expanded *TRDV4* T cells might lead to relatively better outcome for patients diagnosed with T – cell acute lymphoblastic leukemia (T-ALL) after HSCT [19]. However, little is known about the correlation between γδ T cells and AML outcome. In this study, we analyze the distribution and clonality of *TRDV* subfamilies in γδ T cells sorted from the peripheral blood (PB) and discuss the clinical relevance of γδ T cell subfamilies in AML patients.

## Results

### Expression frequency and clonality of TCR *V*δ T cells in AML

**Fig. 1:**
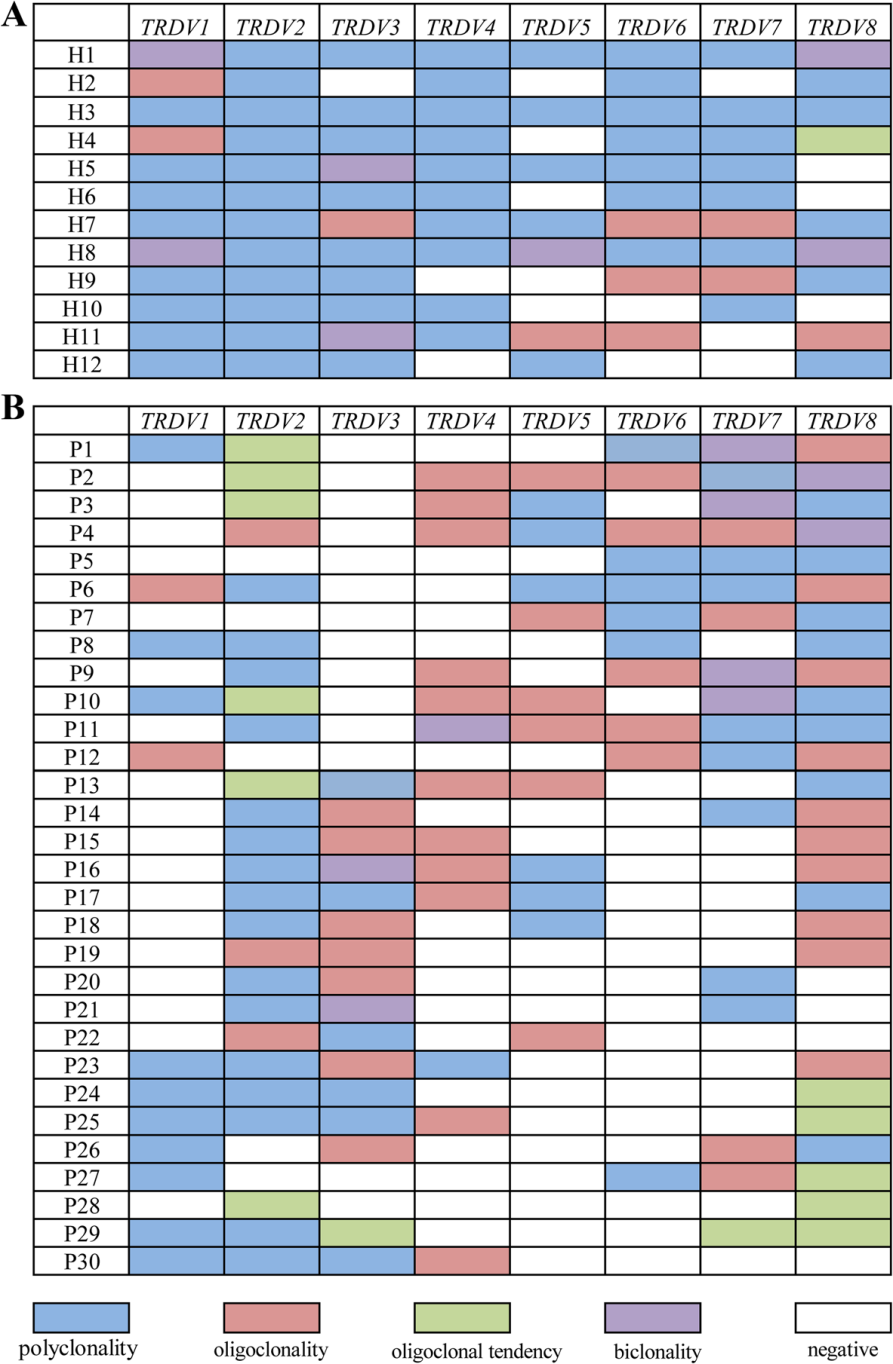
Distribution and clonality of the *TRDV* subfamilies in γδ T cells. **a** 12 healthy individuals. **b** 30 AML patients

**Fig. 2:**
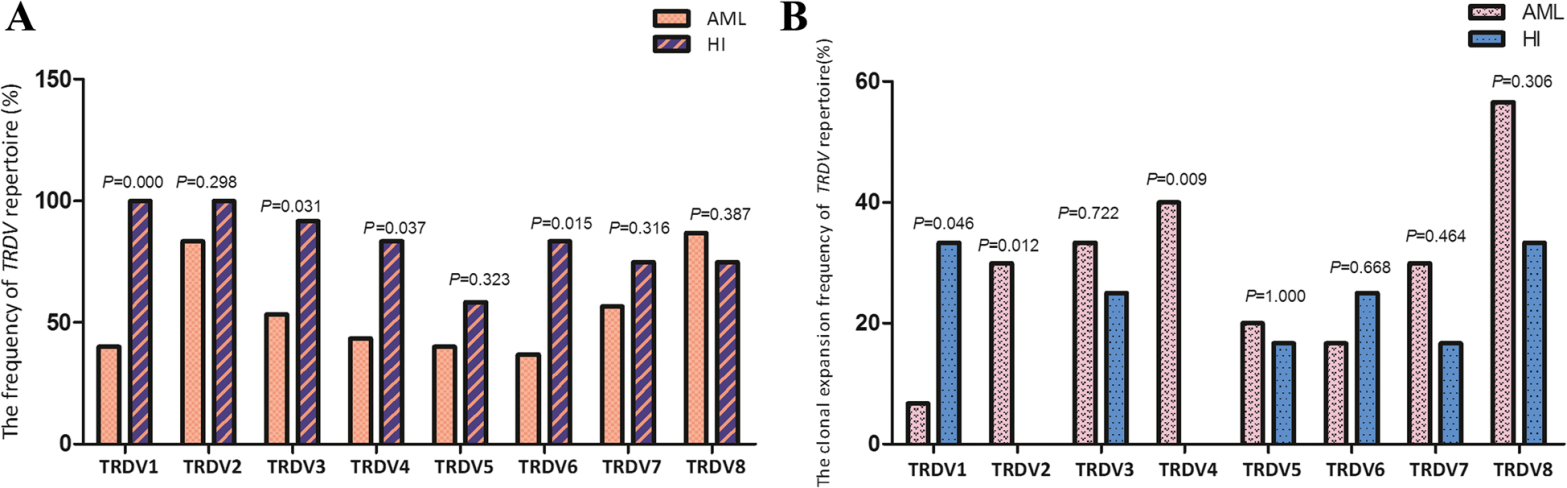
Frequencies of the *TRDV* subfamilies in γδ T cells from AML patients and healthy individuals (using the Fisher’s exact test). **a** Expression frequencies. **b** Clonal expansion frequencies

In this study, the complementarity-determining region 3 (CDR3) sizes of eight *TRDV* subfamily genes were analyzed in γδ T cells sorted from peripheral blood mononuclear cells (PBMCs) from 30 patients with AML and 12 healthy individuals using RT-PCR and GeneScan (Fig. 1). Approximately, 25–75 % of the *TRDV* subfamilies were expressed in 30 different AML patients. The mean value of the number of expressed *TRDV* subfamilies was 4.40 ± 1.07, which was significantly lower than that in healthy individuals (6.67 ± 1.23, *P* = 0.000). The most frequently expressed subfamilies in the AML patients were *TRDV8* (26/30; 86.67 %) and *TRDV*2 (25/30; 83.33 %). *TRDV*6 was detected in only 11 patients (11/30; 36.67 %), and the frequencies of *TRDV1*, *TRDV3*, *TRDV4*, and *TRDV6* were significantly lower than those in healthy individuals (*P* = 0.000, 0.031, 0.037, and 0.015, respectively) (Fig. 2a).

The majority of the *TRDV* subfamilies in the γδ T cells displayed polyclonal expansion with a Gaussian distribution of CDR3 lengths (multi-peaks) corresponding to a polyclonal rearrangement pattern. PCR product analysis produced a single dominant peak or double peaks, which demonstrate a skewed spectratype profile termed “oligoclonality” or “biclonality”, respectively. “Oligoclonality trending” is a classification with a profile between that of polyclonality and oligoclonality [19]. Clonal expansion was detected for all eight *TRDV* subfamilies in the γδ T cells. Greater than two *TRDV* subfamilies demonstrated oligoclonality, biclonality, or oligoclonality trending in all of the AML samples. In addition, the oligoclonally expanded γδ T cells were distributed in almost all of the *TRDV* subfamilies in the AML patients with the exception of *TRDV*1 (6.67 %, 2/30), and the most frequently oligoclonally expanded *TRDV* subfamilies were *TRDV8* (17/30, 56.67 %) and *TRDV4* (12/30, 40 %). The clonal expansion frequencies of the *TRDV2* and *TRDV4* subfamilies were significantly higher than those in healthy individuals (*P* = 0.012 and *P* = 0.009); however, a significantly lower clonal expansion frequency for *TRDV1* was observed in the AML patients (*P* = 0.046) (Fig. 2b).

### Clinical relevance of the oligoclonal expansion of TCR *V*δ T cells in AML

The association between AML outcome, the clonality of *TRDV* subfamilies in γδ T cells, age, WBCs, blast cell percentage in PB, and the absolute number of γδ T cells in PB was analyzed by multivariate non-conditional logistic regression analysis and multivariate stepwise regression analysis. The results demonstrated that oligoclonal expansion of the *TRDV4* and *TRDV8* subfamilies are independent protective factors (odds ratio (OR) = 0.137, 95 % confidence interval (CI) 0.015–1.210; OR = 0.067, 95 % CI 0.005–0.843), and the percentage of blast cells in PB was an independent risk factor for complete remission (CR) (OR = 1.047, 95 % CI 1.009–1.087).

**Fig. 3:**
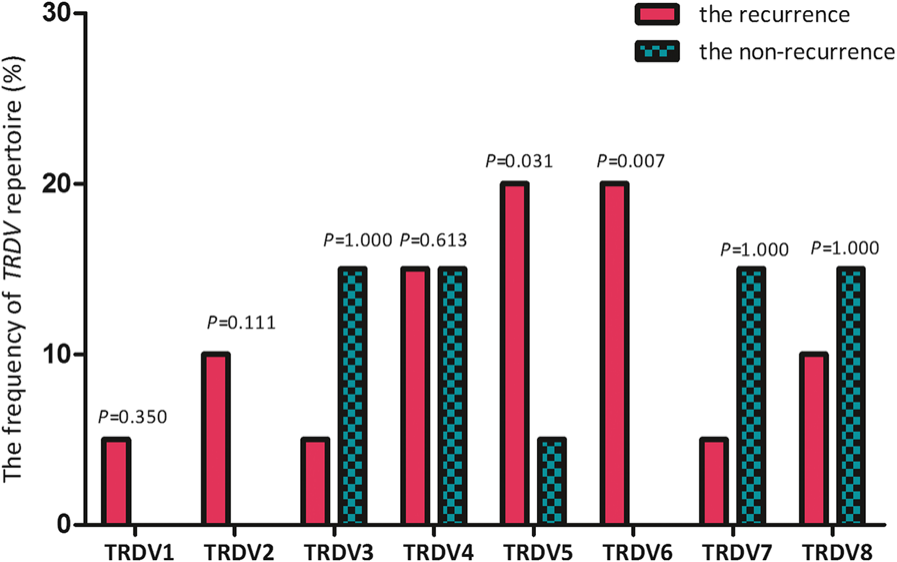
Oligoclonal expansion frequencies of the *TRDV* subfamilies in γδ T cells from AML patients (using the Fisher’s exact test)

**Fig. 4:**
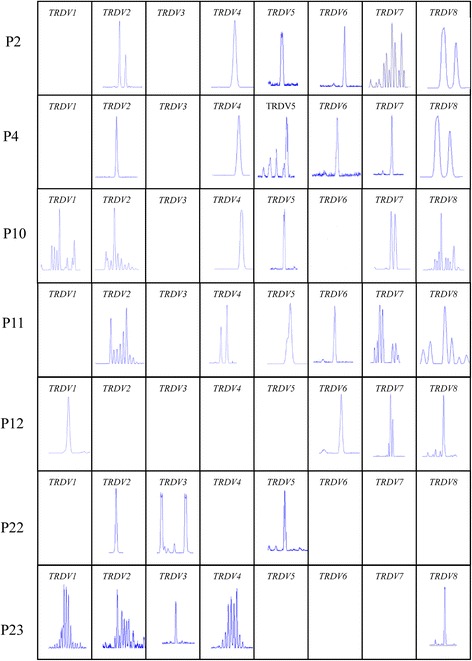
GeneScan results of the *TRDV* subfamily members in γδ T cells from AML cases with recurrence

We also observed that seven patients underwent relapse after achieving CR. In addition, we compared differences in the oligoclonal expansion of *TRDV* subfamilies between those with recurrence and those with non-recurrence. Interestingly, the oligoclonal expansion frequencies of *TRDV5* and *TRDV6* in the recurrence group were significantly higher than those in the non-recurrence group (*P* = 0.031 and *P* = 0.007) (Figs. 3 and 4). Logistic regression analysis demonstrated that oligoclonal expansion of *TRDV5* and *TRDV6* was an independent risk factor for AML recurrence (OR = 21.822, 95 % CI 1.426–333.877; OR = 44.603, 95 % CI 2.169–917.358, respectively).

## Discussion

Although treatments for curing AML have appeared in recent years, the clinical outcomes of some AML patients have not been positive. Recent studies have suggested that there were restricted distribution and clonality for the *TRDV* subfamilies in different diseases including immune thrombocytopenic purpura, B cell non-Hodgkin lymphoma, allergic rhinitis, MDS, CML, and graft versus host disease (GVHD) [20–25]. Understanding the mechanisms underlying the γδ T cell immune response in patients with leukemia is vital for developing strategies for leukemia immunotherapy [26–28]. To investigate the immune characteristics of γδ T cells in patients with AML, we first sorted the γδ T cells from the PB of AML patients and analyzed their TCR *V*δ repertoire. We then attempted to characterize the correlation between oligoclonal expansion of TCR *V*δ repertoire and clinical outcome.

In PB T cells from healthy individuals, the *TRDV* repertoire expression pattern is unrestricted. In contrast, we found significantly restricted *TRDV* subfamily expression in the γδ T cells from patients with AML. Such an alteration in the *TRDV* repertoire distribution in AML appeared to be different for different diseases, e.g., the most frequently expressed *TRDV* genes were *TRDV1* and *TRDV2* followed by *TRDV8* and *TRDV3* in MDS patients [22]. This observation suggests that different subfamilies of γδ T cells might be preferentially active in different diseases and different immune statuses for patients with the same disease.

In immunodeficient patients with leukemia, it is difficult to distinguish the role of oligoclonal T cells, which may serve as reactive T cell clones directed against leukemia. In contrast, there may be clonal absence because T cell proliferation is suppressed by different factors in leukemia. For example, *TRDV2* T cells are reduced and dysfunctional in some MDS patients [15, 19, 29]. To further investigate the role of oligoclonal *TRDV* T cells in AML patients, we first analyzed the correlation between clonally expanded *TRDV* T cells and clinical outcome. We found that different oligoclonal *TRDV* subfamily T cells might have unique functions. We found that the clonal expansion patterns of *TRDV4* and *TRDV8* T cells might be independent protective factors for CR, which is consistent with our previous findings in which we found that clonally expanded *TRDV4* T cells might be related to better outcome for a T-ALL patient [19]. We suggested that such expanded *TRDV4* and *TRDV8* T cell clones might be reactive T cell clones directed against leukemia that serve as biomarkers for the therapeutic efficacy of AML patients. However, a higher frequency of clonally expanded *TRDV8* was also found in MDS patients who developed AML [22]. Thus, further investigation is needed to characterize the function of *TRDV8* T cell clones in vitro and in vivo. Interestingly, we also found that *TRDV5* and *TRDV6* T cells might be related to AML recurrence. These oligoclonal *TRDV5* and *TRDV6* T cells might be indicators of minimal residual disease in AML patients. However, this hypothesis requires confirmation with a larger cohort.

In conclusion, to the best of our knowledge, this is the first attempt to analyze the distribution and clonality of the *TRDV* repertoire in γδ T cells in AML patients. Alterations in the peripheral *TRDV* gene repertoire are an important characteristic of γδ T cells in AML patients, which may be related to the immune response, antileukemia effects, and patient outcome. These findings might provide new data regarding the characteristics of cellular immunity in AML patients. The oligoclonal expansion of TCR *V*δ T cells may serve not only as an immune biomarker for clinical outcome but also as an antileukemia immune status indicator in AML patients. Based on this study, we will further investigate the function of the TCR *V*δ T cells subfamilies in co-culture models and mouse xenograft model.

## Materials and methods

### Samples

After obtaining patient consent, PBMCs from 30 AML patients (17 males and 13 females, median age 33 years, range 17–67 years) was collected. The diagnosis of AML was based on the French–American–British (FAB) criteria: 6 patients were classified as M0, 1 patient was M1, 5 patients were M2, 10 patients were M3, 3 patients were M4, and 5 patients were M5. Twelve healthy individuals (5 males and 7 females, median age 41 years, range 29–62 years) served as the control group. The clinical data of the patients are listed in Additional file 1: Table S1. This study was approved by the Ethics Committee of the Medical School of Jinan University of Guangdong Province in China, and all procedures were conducted according to the guidelines of the Medical Ethics Committees of the Health Bureau of the Guangdong Province of China.

### γδ T cell sorting

The γδ T cells in the PB from 30 AML patients and 12 healthy individuals were sorted by using γδ monoclonal antibodies and the MACS magnetic cell sorting technique (Miltenyi Biotec, Bergisch Gladbach, Germany) [30].

### RNA isolation and cDNA synthesis

RNA was extracted from the sorted γδ T cells using TRIzol RNA extraction buffer according to the manufacturer’s protocol (Invitrogen, Carlsbad, CA, USA). The quality of the RNA was analyzed in a 1.5 % agarose gel stained with ethidium bromide. Two micrograms of RNA was reverse transcribed into first-strand complementary DNA (cDNA) with random hexamer primers using the reverse transcriptase of the SuperScript II Kit (Gibco, Gaithersburg, MD, USA). The cDNA quality was confirmed by RT-PCR of the β_2_ microglobubin (β_2_M) gene [31].

### *TRDV* subfamily expression analysis by RT-PCR

Eight sense *TRDV* sense primers and a single TRDC reverse primer were used in unlabeled PCR to amplify the *TRDV* subfamilies. Subsequently, runoff PCR was performed with fluorescent primers labeled at the 5' end with a FAM fluorophore (Cδ-FAM), which was purchased from TIB MOLBIOL GmbH, Berlin, Germany. The sequences of the primers are listed in Additional file 2: Table S2. PCR was performed as previously described [22]. The cDNA aliquots (1 μl) were amplified in 20 μl reactions using one of the eight *V*δ primers and a Cδ primer. The final reaction mixture contained 0.5 μM sense and antisense primers, 0.1 mM dNTPs, 1.5 mM MgCl_2_, 1× PCR buffer, and 1.25 U Taq polymerase (Promega, Foster City, CA, USA). Amplification was performed with a thermal cycler (BioMetra, Germany). After a 3-min denaturation at 94 °C, 40 cycles of 94 °C for 1 min, 60 °C for 1 min, and 72 °C for 1 min were performed followed by a final 6-min elongation at 72 °C. The products were then stored at 4 °C [32].

### *TRDV* subfamily clonality identification by GeneScan analysis

Aliquots of unlabeled PCR products (2 μl) were subjected to a runoff reaction cycle using a fluorophore-labeled Cδ-FAM primer. The labeled runoff PCR products (2 μl) were heat-denatured at 94 °C for 4 min with 9.5 μl formamide (Hi-Di Formamide, ABI, USA) and 0.5 μl size standards (GENESCAN™-500-LIZ™, Perkin Elmer, ABI). The samples were then loaded in a 3100 POP-4™ gel (Performance Optimized Polymer-4, ABI, USA) and resolved by electrophoresis with a 3100 DNA sequencer (ABI, PerkinElmer) for size and fluorescence intensity determination using GeneScan software [33–35].

### Statistical analysis

All data analyses, including statistical calculations and graphical displays, were performed using SPSS 13.0 and GraphPad software. Univariate analysis was performed using the Mann–Whitney test to compare the means of the expression of the clonally expanded *TRDV* subfamilies between AML patients and healthy individuals. Different frequencies of *TRDV* subfamilies were compared using Fisher’s exact test. Oligoclonal *TRDV* expansion differences between the recurrence and non-recurrence groups were measured using the Fisher’s exact test. Binary logistic regression analysis was performed to determine associations between the clonal expansion of γδ T cells and the outcome of the AML patients. All analyses included the following variables: γδ T cell clonal expansion, age, WBC count, percentage of blast cells in PB, absolute number of γδ T cells in PB, and clinical status. Odds ratios and 95 % confidence intervals were also calculated. Only values with *P* < 0.05 were considered statistically significant.

## Additional files

Additional file 1: Table S1. AML patient characteristics. (DOCX 15 kb)
Additional file 2: Table S2. List of primer sequences used for the *TRDV* subfamilies. (DOCX 13 kb)

## Abbreviations

AML: Acute myeloid leukemia
CDR3: Complementarity-determining region 3
CI: Confidence intervals
CML: Chronic myeloid leukemia
CR: Complete remission
FAB: French–American–British
GVHD: Graft versus host disease
HSCT: Hematopoietic stem cell transplantation
MDS: Myelodysplastic syndromes
MM: Multiple myeloma
OR: Odds ratio
PB: Peripheral blood
PBMCs: Peripheral blood mononuclear cells
T-ALL: T cell acute lymphoblastic leukemia
TCR: T cell receptor
β_2_M: β_2_ microglobubin
