# Supplementary material for: Oligoclonal expansion of TCR Vδ T cells may be a potential immune biomarker for clinical outcome of acute myeloid leukemia
Source: J Hematol Oncol. 2016 Nov 18;9:126. doi: 10.1186/s13045-016-0353-3 (PMC5116135; doi:10.1186/s13045-016-0353-3)
Supplement: Additional file 1: Table S1. — AML patient characteristics. (DOCX 15 kb) [file 13045_2016_353_MOESM1_ESM.docx]

**Supplement Table 1 AML patient characteristics**

| NO. | Gender | | Age | Diagnosis | | WBC  (10^9^/L) | | RBC  (10^9^/L) | | PLT  (10^9^/L) | | Blast cells (%) | | Hb  (g/L) | | Disease status | | Relapse |
| --- | --- | --- | --- | --- | --- | --- | --- | --- | --- | --- | --- | --- | --- | --- | --- | --- | --- | --- |
| P1  P2  P3  P4  P5  P6  P7  P8  P9  P10  P11  P12  P13  P14  P15  P16  P17  P18  P19  P20  P21  P22  P23  P24  P25  P26  P27  P28  P29  P30 | F  M  F  F  M  F  M  M  F  F  M  F  M  F  M  F  M  M  M  M  F  F  M  F  M  M  F  M  M  M | 48  54  23  23  30  68  35  17  18  49  17  34  67  54  36  47  23  29  68  35  34  33  77  42  41  30  30  50  44  48 | | M2  M0  M0  M4  M2  M0  M3  M3  M3  M5  M5  M5  M2  M3  M4  M3  M4  M3  M0  M0  M2  M0  M2  M3  M5  M3  M3  M1  M5  M3 | 266.08  2.59  7.03  54.04  7.25  30.18  23.1  5.6  31.84  2.86  13.56  5.51  54.89  1.12  64  2.2  59.54  80.4  28.32  21.11  28.73  50.64  28.24  14.53  14.48  2.33  5.16  135.81  94.19  6.51 | | 2.27  3.56  4.64  2.12  2.33  1.86  3.03  4.24  2.77  2.77  2.31  4.28  3.32  1.47  3.98  2.5  2.45  3.58  2.54  1.28  3.89  3.78  2.31  2.55  2.2  3.54  2.32  1.57  3.47  2.38 | | 86  222  89  39  24  49  20  104  14  58  49  61  12  20  36.5  51  24.4  38  40  12  27  118  29  21.2  9.4  31  16  9  89  12 | | 21  25  15  5  32  92  87  70  75  10  93  7  93  5.5  24  6.2  4.5  4  24  76  43  44  75  60  87  22  73  93  62  63 | | 63  101  83  64  69  62  87  121  75  82  71  136  106  54  118  75  78.5  109  76  44  133  111  73  81  62.6  116  67  47  112  111 | | PR  CR  CR  CR  CR  NR  CR  CR  NR  CR  CR  CR  NR  CR  PR  CR  CR  CR  NR  NR  CR  CR  CR  CR  NR  CR  CR  CR  PR  NR | | YES  YES  YES  YES  YES  YES  YES | |

Notes: WBC: white blood cell, RBC: red blood cell, PLT: platelet, Hb: hemoglobin, F: female, M: male, M0: minimally differentiated AML, M1: AML without maturation, M2: AML with maturation, M3: acute promyelocytic leukemia, M4: acute myelomonocytic leukemia, M5: acute monocytic leukemia, CR: complete remission, PR: partial remission, NR: no remission
