# Supplementary material for: Oligoclonal expansion of TCR Vδ T cells may be a potential immune biomarker for clinical outcome of acute myeloid leukemia
Source: J Hematol Oncol. 2016 Nov 18;9:126. doi: 10.1186/s13045-016-0353-3 (PMC5116135; doi:10.1186/s13045-016-0353-3)
Supplement: Additional file 2: Table S2. — List of primer sequences used for the TRDV subfamilies. (DOCX 13 kb) [file 13045_2016_353_MOESM2_ESM.docx]

**Supplement Table 2 List of primer sequences used for the *TRDV* subfamiies**

| Primer | | Sequence |  |
| --- | --- | --- | --- |
| VD1  VD2  VD3  VD4  VD5  VD6  VD7  VD8  Cδ  Cδ-FAM | 5’-GTGGTCGCTATTCTGTCAACT-3’  5’-GCTCCATGAAAGGAGAAGCGA-3’  5’-CACTGTATATTCAAATCCAGA-3’  5’-TGACACCAGTGATCCAAGTTA-3’  5’-TCTGCACATTGTGCCCTCCCA-3’  5’-TATCATGGATTCCCAGCC-3’  5’GAACATCACAGCCACCCAGACCG-3’  5’-ACTTCCAGAAAGCAGCCAAA-3’  5’-AACAGCATTCGTAGCCCAAGCAC-3’  5’-FAMGTTTATGGCAGCTCTTTGAAGGT-3’ | |  |
